# Complete Genome Sequencing of 10 Brucella abortus Biovar 3 Strains Isolated from Water Buffalo

**DOI:** 10.1128/genomeA.00180-18

**Published:** 2018-04-19

**Authors:** Rubina Paradiso, Massimiliano Orsini, Daniela Criscuolo, Rosanna Borrelli, Ornella Valvini, Cesare Cammà, Maria Luisa Chiusano, Giorgio Galiero, Giorgia Borriello

**Affiliations:** a Istituto Zooprofilattico Sperimentale del Mezzogiorno, Portici, Italy; b Istituto Zooprofilattico Sperimentale G. Caporale, Teramo, Italy; c Dipartimento di Agraria Università degli Studi di Napoli Federico II, Portici, Italy

## Abstract

Brucellosis is a zoonotic disease that affects both humans and animals. Its distribution is global, concentrated in the Mediterranean area, India, Central Asia, and Latin America. Here, we present a complete genome assembly of 10 Brucella abortus strains isolated from water buffaloes farmed in the Campania region of Italy.

## GENOME ANNOUNCEMENT

Brucellosis is one of the most widespread bacterial zoonotic diseases in the world, affecting both humans and domestic and wild animals (1). Animal brucellosis is responsible for many economic losses due to abortions, loss of production (mainly reduced milk production), viable but weak calves, and reproductive disorders.

Over the years, several studies based on the identification and biotyping of field strains of Brucella were carried out to better understand the epidemiology of brucellosis, identify appropriate antigens, manage disease outbreaks, and set up efficient prevention and control programs (2). However, for a better understanding of the molecular mechanisms associated with Brucella virulence, phenotypic and genotypic characterization of circulating strains is required. Therefore, we selected 10 B. abortus bv. 3 strains, isolated from water buffalo farmed in the Campania region of Italy, where this pathogen is still present (3), for genetic characterization by sequencing analysis.

Strains were inoculated onto Brucella medium base (Oxoid) and incubated at 37°C in 5 to 10% CO_2_. DNA was purified with the QIAamp DNA minikit (Qiagen), as described by the manufacturer. For sequencing analysis, approximately 50 to 100 ng of genomic DNA were used to produce fragments of 400 bp in length. Libraries were prepared using an Ion Shear Plus reagents kit (Life Technologies) for the Ion Torrent sequencing platform. Sequencing reads were checked for quality using FastQC, and low-quality sequences were removed using PRINSEQ lite. High-quality sequences were successfully assembled using SPAdes (4) version 3.8.2 software, improved with Pilon version 1.8, and manually checked to close eventual gaps. Draft assemblies of the 10 strains were annotated using the NCBI Prokaryotic Genome Annotation Pipeline version 4.2.

The Brucella genome has two circular chromosomes. For each strain, 95.08% ± 3.12% of the obtained sequences was assembled, with a mean contig number among assemblies of 36% ± 3.73%. For each strain, we identified all coding genes and genes coding for proteins involved in virulence mechanisms. Using Bowtie2 version 2.2.9, reads were aligned to the genome sequence of the reference strain B. abortus A13334 (GenBank accession numbers NC_016795 and NC_016777). The number of single-nucleotide polymorphisms (SNPs) was determined by MUMmer version 3.0. This study highlights the complexity of the Brucella genome and represents a basis for further studies aiming to provide a better comprehension of the virulence mechanisms of this pathogen.

### Accession number(s).

The whole-genome sequences of B. abortus bv. 3 strains were deposited in GenBank under BioProject number PRJNA400357, under the accession numbers listed in Table 1.

**TABLE 1:** Main characteristics and GenBank accession numbers of the 10 assembled B. abortus bv. 3 genome sequences

Strain	No. of genes	*N*_50_	No. of virulence genes	GenBank accession no.	
				Chromosome 1	Chromosome 2
72871	3,669	382	63	CP023239	CP023240
69103	3,377	290	63	CP023225	CP023226
67761	3,157	260	64	CP023223	CP023224
57750	3,280	276	64	CP023221	CP023222
49188	3,233	287	64	CP023217	CP023218
38127	3,298	342	63	CP023237	CP023238
21630	3,383	337	64	CP023235	CP023236
21614	3,745	379	64	CP023233	CP023234
15500	3,523	312	62	CP023231	CP023232
149279	3,231	293	64	CP023227	CP023228
